# Severe Prekallikrein Deficiency Associated with Low Level of Factor XII: A Case Report

**DOI:** 10.30699/IJP.2020.131638.2463

**Published:** 2021-05-09

**Authors:** Massoumeh Shahbazi, Minoo Ahmadinejad, Shahnaz Fakhrzadegan

**Affiliations:** 1 *Blood Transfusion Research Center, High Institute for Research and Education in Transfusion Medicine, Tehran, Iran*; 2 *Department of Hematology and Oncology, School of Medicine, Iran University of Medical Science, Tehran, Iran.*

**Keywords:** Factor XII deficiency, Prekallikrein deficiency, Prolonged aPTT

## Abstract

Hereditary deficiency of plasma prekallikrein (PPK) is a rare autosomal recessive disease. The affected patients are often asymptomatic and diagnosed incidentally during preoperative investigations or during hospitalization by isolated prolongation of activated partial thromboplastin time (aPTT). In this article, we report, a 46-year-old woman who was candidate for two invasive procedures (thyroid FNA and hysterectomy) and underwent preoperative evaluation. Due to prolonged aPTT with normal PT she was referred to the IBTO reference coagulation laboratory for specific coagulation assays. Ultimately, the examinations revealed severe PPK deficiency (<1%) with partial deficiency of factor XII level (25%).

## Introduction

Prekallikrein deficiency was first described in 1965 by Hathaway *et al.* in a large Kentucky family. It was called as "Fletcher factor". In 1973, Wuepper *et al.* further investigated this unknown factor and they called it as “Prekallikrein” (PK). The gene related to PK is KLKB1, which is located on the q34-q35 region of the long arm of chromosome 4 (1). The PPK deficiency is a rare hereditary disorder that causes an isolated marked prolongation of aPTT but is not associated with bleeding tendency. The most important consideration for PPK deficiency is to be diagnosed timely and correctly for preventing any unnecessary transfusion of blood products during or before invasive procedures (1-3). According to the literature, few cases of PPK deficiency have experienced some venous or arterial thrombosis or excessive bleedings after surgery, however, it is not clear whether these symptoms are related to PPK deficiency or not, because other known related risk factors have also been detected in the affected patients. Various diseases have been described in the patients with PPK deficiency as comorbidities including cardiovascular disease, hypertension, thrombosis, stroke, hemorrhage, and systemic lupus erythematous; but it is difficult to confirm their association (4, 5). 

The reported number of PPK deficiency cases in the literature so far is much less than actual number. For at least 15 years, HMWK and PPK tests have been performed in the special Coagulation Lab of Iranian Blood Transfusion Organization (IBTO) for all the patients who have prolonged aPTT but normal intrinsic coagulation factors and without evidence of coagulation inhibitors. During this period of time this patient was the second case of PPK deficiency that was diagnosed.

## Case Report

A 46-year-old woman with a history of hypothyroidism has been a candidate for thyroid FNA (fine needle aspiration) to evaluate thyroid cold nodules and hysterectomy for uterine fibroids. During preoperative work up, an isolated prolongation of aPTT was noted and the patient was referred for further evaluation to the special coagulation laboratory of IBTO with suspicion to Lupus Anticoagulant (LA). The patient had a history of easy bruising and also bleeding after one tooth extraction (she had 4 tooth extractions, which in the last one needed to be sutured to stop the bleeding). She also had two previous surgeries (appendectomy and cesarean section) without any abnormal bleeding. According to the ISTH-SSC Bleeding Assessment Tool the total bleeding score was 5 (cutaneous: 1, bleeding from minor wounds: 1, oral cavity: 1, tooth extraction: 2). The patient’s parents did not have any consanguinity. She had two sisters and three brothers, as well as a 16-year-old girl who were rejected for any history of bleeding or thrombotic events. She had a history of long-term hypothyroidism and resistant to routine doses of thyroid hormone. Initial laboratory tests in IBTO showed: HGB: 12.7 g/dL (12-16), RBC: 4.86 × 10^6^/UL (3.3-5.8), WBC: 6.6 × 10^3^/UL (3.5-11), platelet: 323 × 10^3^/UL, aPTT: 206 seconds, and PT: 10 seconds (see Table 1). Special coagulation assays revealed normal level of all factors except PPK (<1%) and factor XII (25%). The LA testing was performed by a screening and confirmatory panel and the results showed no evidence of antiphospholipid antibody (Table 1). Finally, the patient was considered as severe prekallikrein deficiency concomitant with partial deficiency of factor FXII.

Routine coagulation assays were performed by the reagents from Instrumentation Laboratory Company and with ACL TOP Autoanalyzer. The PPK test was done by Technocolone PK reagent and all of the LA panel tests were done by STAGO reagents and STAR Max Autoanalyzer (STAGO France).

**Table 1:** Laboratory tests results released byn IBTO

Reference ranges	Unit	Results	Test
			
**9-12.5**	Second	10	**PT**
**28-38**	Second	206	**PTT**
**Up to 38**	Second	32	**Mixed aPTT**
**13-16**	Second	14.3	**TT**
		Negative	**Lupus Anticoagulant**
**31-47**	Second	> 70	**PTT LA**
**Up to 47**	Second	45	**Mixed PTT LA**
**32-46**	Second	36.4	**dRVVT screen**
**31-40**	Second	33.3	**dRVVT confirm**
**< 1.2**	Ratio	1.1	**dRVVT Ratio**
**50-150**	IU/dL	172	**F VIII:C**
**65-150**	IU/dL	89	**FIX**
**65-150**	IU/dL	71	**FXI**
**50-150**	IU/dL	25	**FXII**
**50-150**	IU/dL	< 1	**Prekallikrein**
**50-150**	IU/dL	144	**VWF Ag**

The result of all coagulation assays were re-confirmed on two separate specimens with one week interval.

## Discussion

Prekallikrein is a glycoprotein mainly synthetized in the liver and secreted into the plasma as a single-chain peptide with a molecular weight of 88,000 Daltons, and normal concentration of 40 mg/mL. About 75-90% of this coagulation factor is combined with HMWK. The activation of prekallikrein results in production of kallikrein, and activation of factor XI and factor XII. Patients with PPK deficiency, which is a rare autosomal recessive disorder (2, 6), typically have isolated prolongation of aPTT and they are often asymptomatic (7). According to the recent study by Barbosa *et al.* the deficiency of contact factors is the second cause of unknown prolonged aPTT after antiphospholipid antibodies (8). Prolonged aPTT with normal PT can also occur in the cases with FVIII, FIX, FXI, FXII, VWF and HMWK deficiencies, heparin treatment, receiving lepirudin or argatroban and in the presence of non-specific inhibitors like lupus anticoagulant (9). Our patient had no deficiency of any above factors and did not mention a history of medication except for thyroid hormone. This patient had been referred to our laboratory due to a prolonged aPTT with the possibility of lupus anticoagulant. In our wide laboratory examinations, a combined severe PPK and mild FXII deficiencies was confirmed. Based on our knowledge, this is the second case of PPK deficiency diagnosed in Iran after the first case that was reported by Shahverdi *et al.* in 2017 (10).

The occurrence of bleeding events in the patients with PPK deficiency is not a common phenomenon and the reported studies about bleeding complications after surgical procedures show a wide range of bleeding tendency from no bleeding to major bleeding events (5-7, 11). Recently, Barco *et al.* (6) in their largest systematic review study about PK deficiency reported 108 cases from 86 families. They investigated these cases for three main clinical presentations: major bleeding events, thromboembolic events and other comorbidities during their life time. They reported 4 major bleeding events in 96 individuals including one spontaneous recurrent hematemesis and three provoked hemorrhages (prevalence of major bleeding events in their study was approximately 4%, the annualized incidence rate 0.1 case/100 person year). Girolami *et al*. (5) in 2019 screened 1966 publications which included 53 patients with PPK deficiency. Nearly, half of them (25 patients) were assigned as asymptomatic and 16 of the 53 patients underwent surgery or dental extractions with no bleeding complications. Only four patients showed bleeding events (7.5%) after surgical procedures. Zhou *et al.* (9) have shown in their review study that PPK deficiency in normal people rarely leads to bleeding events. Our patient had no experience of any bleeding complications during her life time except for easy bruising and also bleeding after one out of four teeth extractions.

Thrombotic events among patients with PPK deficiency were reported by several studies (2, 4, 6). Barco *et al.* (6) reported 14.7% prevalence of thrombotic events (14 patients) among 95 patients, which included ischemic strokes, myocardial infarction and venous thromboembolic events. Girolami *et al*. (4) in 2010 reviewed 75 patients with PPK deficiency and reported 9 (12.2%) thrombotic events (both arterial and venous thrombosis). Another study performed by the same author showed that these patients often have concomitant acquired or congenital thrombophilic risk factors (12). Our case did not experience any thrombotic events during her life despite the concomitant deficiency of two contact factors.

The first case of concurrent PPK and factor XII deficiencies was reported in 1981 by Man-Chiu Poon *et al.* (13). They described a 7-year-old boy who had an 18-months history of recurrent epistaxis, without other bleeding manifestations. He was completely normal on physical examination. In the large study of Barco *et al*. (6) one quarter of 111 cases with PPK deficiency had factor XII clotting activity below the reference range. We also found partial deficiency of factor XII (25%) in our case.

There are some sporadic reports about PPK deficiency with some comorbidities e.g. autoimmune diseases including Graves' disease and systemic lupus erythematous. In the adults with multiple comorbidities or systemic inflammation, the effect of PPK deficiency remains controversial and further researches are needed to confirm the causative association between them (14-16). Our case had hypothyroidism and the relation between hypothyroidism and PPK deficiency has not been reported before. However, various studies have shown that hyperthyroidism and hypothyroidism modify the coagulation-fibrinolysis balance and these conditions lead to the increased risk of thrombosis and bleeding events, respectively (17, 18). Hypothyroidism also can increase the incidence of abnormal vaginal bleeding but the evidence to prove this phenomenon is rare while it may be a direct effect of thyroid hormone on muscle contraction. It now appears that low levels of free thyroxin hormone (FT4) can lead to menorrhagia and women with overt hypothyroidism may have menstrual disturbances (3). Our patient had also menstrual disturbances without any increase in bleeding volume for the last year. Although patients with hypothyroidism may have acquired von Willebrand disease (19), the VWF:Ag level was normal in our patient.

The data on the preoperative management of patients with PPK deficiency are also limited. Unal and his colleagues in their literature review on the surgical management of PPK deficient patients observed that most of these patients needed no FFP transfusion and FFP transfusion may be used only in patients with PPK deficiency undergoing invasive procedures (20). Our patient also had two previous surgeries (appendectomy and cesarean section) without any abnormal bleeding or any need for the FFP transfusion. 

## Conclusion

In this study, we presented a case of severe PPK deficiency with concurrent partial factor FXII deficiency that experienced occasional non-significant hemorrhages without any thrombotic events. It seems that PPK deficiency cases are under-diagnosed due to the lack of significant hemorrhagic or thrombotic symptoms in general and low availability of its specific laboratory test in our country. The most significant presentation of these cases is incidental finding of isolated prolongation of aPTT especially in preoperative hemostatic evaluations. Physicians should be aware of this rare congenital deficiency by conducting special coagulation assays and excluding other causes of prolongation of aPTT in order to prevent unnecessary interventions like transfusion of blood products in these patients.
